# Possible strategies for use of artificial intelligence in screen-reading of mammograms, based on retrospective data from 122,969 screening examinations

**DOI:** 10.1007/s00330-022-08909-x

**Published:** 2022-06-15

**Authors:** Marthe Larsen, Camilla F. Aglen, Solveig R. Hoff, Håkon Lund-Hanssen, Solveig Hofvind

**Affiliations:** 1 Section for Breast Cancer Screening, Cancer Registry of Norway, Oslo, Norway; 2 Department of Radiology, Ålesund Hospital, Møre og Romsdal Hospital Trust, Ålesund, Norway; 3 Department of Circulation and Medical Imaging, Norwegian University of Science and Technology, Trondheim, Norway; 4 Department of Radiology and Nuclear Medicine, St. Olavs Hospital, Trondheim University Hospital, Trondheim, Norway; 5 Department of Health and Care Sciences, Faculty of Health Sciences, The Arctic University of Norway, Tromsø, Norway

**Keywords:** Artificial intelligence, Mass screening, Breast neoplasm, Workload, Mammography

## Abstract

**Objectives:**

Artificial intelligence (AI) has shown promising results when used on retrospective data from mammographic screening. However, few studies have explored the possible consequences of different strategies for combining AI and radiologists in screen-reading.

**Methods:**

A total of 122,969 digital screening examinations performed between 2009 and 2018 in BreastScreen Norway were retrospectively processed by an AI system, which scored the examinations from 1 to 10; 1 indicated low suspicion of malignancy and 10 high suspicion. Results were merged with information about screening outcome and used to explore consensus, recall, and cancer detection for 11 different scenarios of combining AI and radiologists.

**Results:**

Recall was 3.2%, screen-detected cancer 0.61% and interval cancer 0.17% after independent double reading and served as reference values. In a scenario where examinations with AI scores 1–5 were considered negative and 6–10 resulted in standard independent double reading, the estimated recall was 2.6% and screen-detected cancer 0.60%. When scores 1–9 were considered negative and score 10 double read, recall was 1.2% and screen-detected cancer 0.53%. In these two scenarios, potential rates of screen-detected cancer could be up to 0.63% and 0.56%, if the interval cancers selected for consensus were detected at screening. In the former scenario, screen-reading volume would be reduced by 50%, while the latter would reduce the volume by 90%.

**Conclusion:**

Several theoretical scenarios with AI and radiologists have the potential to reduce the volume in screen-reading without affecting cancer detection substantially. Possible influence on recall and interval cancers must be evaluated in prospective studies.

**Key Points:**

• *Different scenarios using artificial intelligence in combination with radiologists could reduce the screen-reading volume by 50% and result in a rate of screen-detected cancer ranging from 0.59% to 0.60%, compared to 0.61% after standard independent double reading*

• *The use of artificial intelligence in combination with radiologists has the potential to identify negative screening examinations with high precision in mammographic screening and to reduce the rate of interval cancer*

## Introduction

Breast cancer is the most frequent cancer and the second most frequent cause of cancer death among women worldwide [1]. Mammographic screening along with improved treatment is shown to reduce breast cancer mortality by 30–40% among screening participants [2].

European Commission Initiative on Breast Cancer suggests using double over single reading of mammograms for early detection of breast cancer in screening programs [3]. The overall performance of a screening program depends on organizational factors, image quality, and performance of the radiologists. Screen-reading is a perceptual task, and the radiologists’ performance might be influenced by reading volume and reading conditions [4]. We have previously shown that 23% of screen-detected cancers have a positive assessment score by only one of the two radiologists in BreastScreen Norway [5], and that 20–30% of the screen-detected and interval cancers are classified as missed in retrospective informed review studies [6, 7]. These findings indicate a need for improvements in mammographic screening programs.

Artificial intelligence (AI) has been introduced in numerous areas of healthcare and is unaffected by reading conditions and subjectivity. In the field of mammographic screening, retrospective studies on AI have shown promising results in the classification of cancers [8–11]. However, the study populations are small and not representative of a regular screening setting (i.e., higher prevalence of cancers than in the usual screening setting), which limits the clinical relevance [12]. Furthermore, there is limited knowledge on how AI scores might affect the radiologists’ interpretation. How the AI results are presented to the radiologists, and the timing might also affect the interpretation in different directions.

AI can be used in different ways in a screening setting, and different AI systems may be designed to be used in a specific setup. For instance, AI can be used as a standalone system to directly select examinations for consensus or recall (replacement), as a triage system where examinations are interpreted by no, one or two radiologists based on the risk score from the AI system, or as one reader in an informed or independent double reading setting. Knowledge of the ideal combination of AI and radiologist is sparse [13]. This is an important aspect to consider prior to planning costly prospective studies and before the implementation of AI in screening programs. Estimations of the screening outcome after the use of AI in combination with radiologists require a large volume of retrospective data that are not used in developing or testing the AI algorithm.

Reduced workload for breast radiologists is an important aspect of using AI in screen-reading. Interpreting screening mammograms is a time-consuming process and about 99.4% of the examinations are determined to have a negative outcome [14]. Furthermore, there is a shortage of breast radiologists in Norway, as well as in Europe, and the potential AI has to reduce the screen-reading volume for the radiologists and the costs for the society are substantial [15].

In this retrospective study, we merged screening outcomes from women attending BreastScreen Norway with AI scores and explored consensus, recall, and cancer detection for different theoretical scenarios of AI and the radiologists in screen-reading.

## Material and methods

This study was based on retrospective image data and screening information collected in BreastScreen Norway, a population-based screening program administered by the Cancer Registry of Norway [14]. The study was approved by the Regional Committee for Medical and Health Research Ethics (13294). The data was disclosed with legal bases in the Norwegian Cancer Registry Regulations of 21 December 2001 No. 47 [16].

### Study setting

In Norway, all women aged 50–69 are offered a biennial two-view mammographic screening of each breast. The standard procedure is independent double reading by breast radiologists [17]. The radiologists’ experience with the interpretation of mammograms varies from newly trained to over 25 years of experience. Each breast is assigned an interpretation score of 1–5 by each radiologist to indicate suspicion of malignancy (1, negative for malignancy; 2, probably benign; 3, intermediate suspicion of malignancy; 4, probably malignant; 5, high suspicion of malignancy). Examinations with an interpretation score of 2 or higher by one or both radiologists are discussed at consensus meetings with at least two radiologists present, and the decision to recall the woman for further assessment is made. The program is described in detail elsewhere [14].

### Study sample

**Fig. 1:**
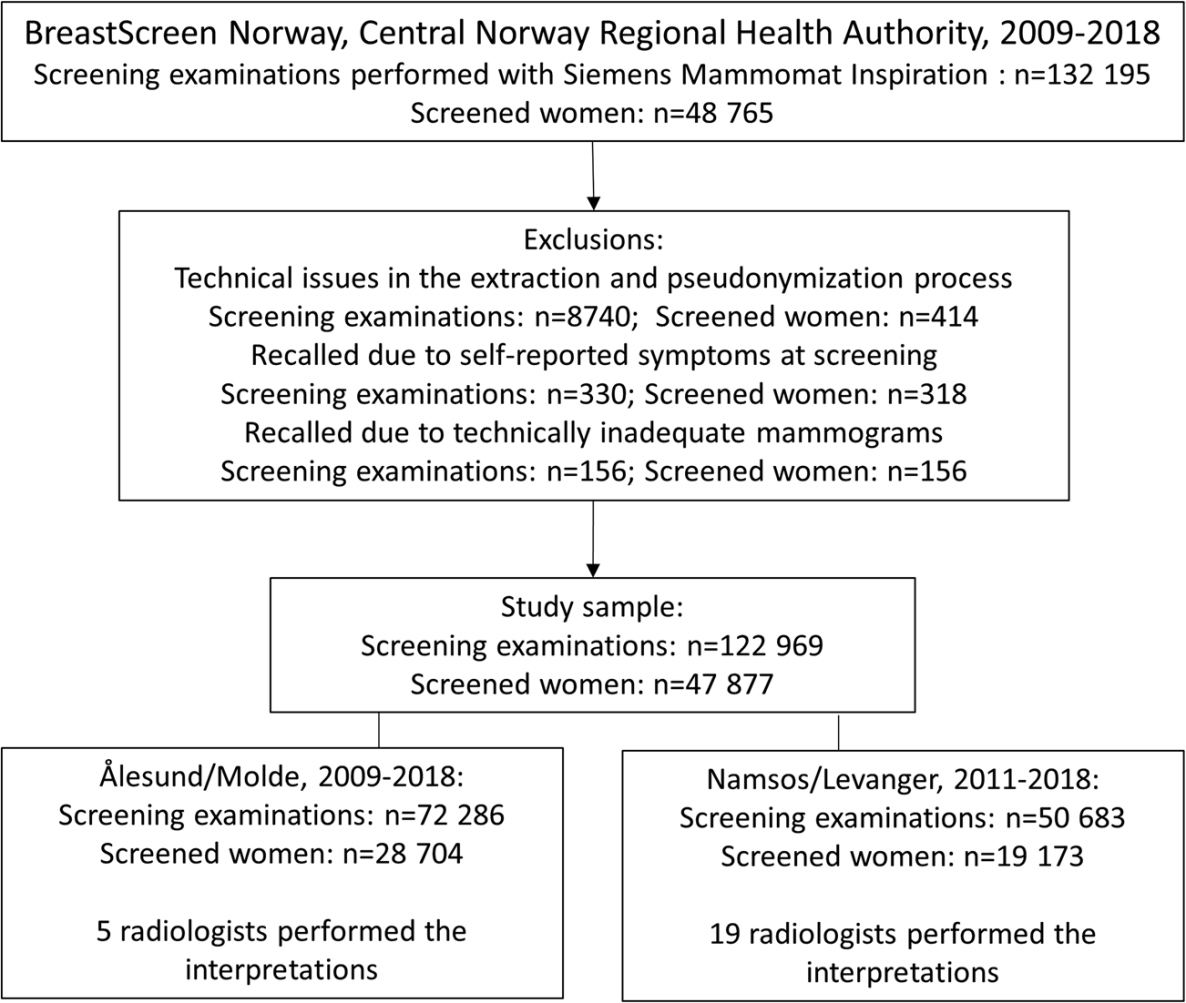
Flowchart of the study sample

A total of 132,195 digital mammographic examinations were performed at four different screening facilities in Central Norway Regional Health Authority during the period from 2009 to 2018. The examinations were interpreted at two breast centers. After exclusions, the final study sample included 122,969 examinations from 47,877 women, resulting in 2–3 screening examinations for each woman (Fig. 1). All examinations were performed using Siemens Mammomat Inspiration. Further details on the study sample and distribution of AI scores are described elsewhere [18].

### The AI system

Pseudonymized examinations were processed with the commercially available AI system *Transpara* version 1.7.0 developed by ScreenPoint Medical. Briefly, the AI system provides one score for each view of each breast based on convolution neural network algorithms. The system is trained on mammograms from different vendors, and results using retrospective data from different vendors are published [9, 19]. In this study, we defined “AI score” for an examination to be the overall exam-level score, which is the highest score of all views. The system aims to distribute the examinations equally across AI scores from 1 to 10, with about 10% of examinations assigned each score. A score of 1 reflects low suspicion of breast cancer, and the risk of breast cancer increases with higher AI scores. In order to make it possible to use more than 10 categories, we also used a continuous “raw AI score”.

The mammograms were processed by the AI system retrospectively, meaning that radiologists did not have access to AI results during the reading process. Results from the AI system were merged with pseudonymized screening information using random study identification numbers after being processed with the AI system. AI scores and retrospective screening information after standard independent double reading performed in a usual screening setting, 2009–2018, were used to estimate possible outcomes for different scenarios of AI and radiologists in screen-reading of mammograms.

### Scenarios of combining AI and radiologists in screen-reading

We defined different theoretical scenarios for how AI score and the radiologists’ interpretation could be combined in screen-reading, and estimated consensus, recall, and cancer detection. Numerous scenarios are possible. We included 11 (Table 1). Results from a real screening setting using independent double reading at the two centers served as the reference. For the different scenarios, we presented volume reduction which represented the reduced number of screening examinations required interpreted by the radiologists. The reduction in reading volume should not be considered the same as a reduction in overall workload as we have not estimated time spent on consensus or screen-reading of the selected examinations, which we expect to differ according to the availability of the AI score or not.

**Table 1:** Definition of different scenarios artificial intelligence (AI) and radiologists can be combined and used in screen-reading. Percentage of screen-readings performed by AI, by the radiologists (R1 and R2) and the corresponding reduction in screen-reading volume are also presented. The volume reduction relates to the screen-reading of mammograms prior to consensus and further assessments. An AI score of 1 indicates the lowest probability of malignancy by the AI system, and 10 indicates the highest probability

Scenarios		Selection rate of AI and radiologists/setup based on AI score	AI, % of screen-readings	R1, % of screen-readings	R2, % of screen-readings	Reduced screening volume, %
	Standard independent double reading	0	0	100	100	0
1	AI as one of two readers	AI and R1 selects 5.8% each*	100	100	0	50
2	AI as one of two readers	AI selects 10.1% (AI score = 10) and R1 selects 5.8%*	100	100	0	50
3	AI selects cases to be double read	AI score 1–5: negative AI score 6–10: R1+R2	100	50	50	50
4	AI selects cases to be double read	AI score 1–7: negative AI score 8–10: R1+R2	100	30	30	70
5	AI selects cases to be double read	AI score 1–9: negative AI score 10: R1+R2	100	10	10	90
6	AI selects cases to be single read	AI score 1–5: negative AI score 6–10: R1	100	50	0	75
7	AI selects cases to be single and double read	AI score 1–5: R1 AI score 6–10: R1+R2	100	100	50	25
8	AI selects cases to be single and double read	AI score 1–7: R1 AI score 8–10: R1+R2	100	100	30	35
9	AI selects cases to be single and double read	AI score 1–5: negative AI score 6–7.5: R1 AI score 7.6–10: R1+R2	100	50	25	63
10	AI selects cases to be single and double read	AI score 1–5: negative AI score 6–7.5: R1+R2 AI score 7.6–10: R1	100	50	25	63
11	AI selects cases to be recalled	3.2%*	100	0	0	100

*5.8% mimics the average selection rate/positive interpretations of the individual radiologists in the study sample, and 3.2% mimics the recall rate in the study sample after independent double reading

In scenario 1, AI was used as one of two readers and 5.8% of the examinations with the highest AI score were selected for consensus by the AI system. The rate of 5.8% was equal to the average rate of positive interpretations by the individual radiologists in the study sample. In scenario 2, AI was also used as one of two readers, but in this scenario, AI selected 10% of the examinations for consensus; 10% corresponded to an AI score of 10. Examinations with an interpretation score of 2 or higher by R1 were also included in the consensus pool in scenarios 1 and 2. In scenarios 3–10, the AI system was used as a triage system and the AI score was used to determine whether examinations should be interpreted by no, one, or two radiologists (Table 1). In scenario 11, the selection rate of AI was set to the recall rate for independent double reading, to explore results with AI as a standalone system.

The retrospective data represented the radiologists’ interpretations in a normal screening setting without AI scores available. Scenarios 1–2 differ somewhat from scenarios 3–11 since AI was used as one of the two readers, and AI could select cases for consensus that radiologists did not select. This approach might imply higher uncertainties in the estimations. However, recalls represented actually recalled women in the study sample after independent double reading for scenarios 1–10. The estimated number of screen-detected cancers was verified screen-detected cancers diagnosed among the recall examinations for the different scenarios. For scenarios 1–10, we presented interval cancers selected for consensus as these are the ones that have the greatest potential to be detected in a prospective screening setting where the AI score would be available at a consensus. We presented the number of examinations selected for consensus that were later diagnosed with interval cancer and calculated the potential maximum rate of screen-detected and reduced rate of interval cancer when including these cases. If signs of the later presenting interval cancer were present at screening (missed interval cancer) and correctly marked with a high AI score, there could be a chance of detecting these cases as screen-detected. In scenario 11, we presented interval cancers that AI selected to be recalled. In the real screening setting, some of these cases were not actually recalled.

### Cancer definition and detection

Screen-detected cancers were defined as breast cancer diagnosed after a recall and within 6 months after the screening examination. Both ductal carcinoma in situ and invasive carcinoma were considered breast cancer. Interval cancers were defined as breast cancers diagnosed within 24 months after a negative screening or 6–24 months after a false-positive screening result [20]. For the interval cancer cases, prior screening mammograms were processed with the AI system. Recall was defined as a screening examination resulting in further assessments due to abnormal mammographic findings.

In the analysis of the sensitivity, for the real setting of independent double reading, screen-detected cancers were considered true positive and interval cancers were considered false negative. For all scenarios, we considered true positive to be (a) screen-detected cancers only and (b) screen-detected and interval cancers where prior screening examination was selected for consensus or recall for scenario 11. In *a*, screen-detected cancers not among recall examinations for the different scenarios and all interval cancers were considered false negatives and in *b*, screen-detected cancers not among recalls and interval cancers not selected for consensus were considered false negatives.

### Statistical analysis

Categorical variables were presented with frequencies and percentages. In the scenarios where AI was used as one reader, we could have combined AI score with radiologist 1 (R1), radiologist 2 (R2), or a random combination of the two from the independent double reading setting to estimate the different rates. Due to independent double reading, we expect similar results for the two readers, but we have presented results for AI and R1 only as the first reader are, by definition, independent. Sensitivity with a 95% confidence interval (CI) was calculated with the logit-transformed formula based on true positives and false negatives as described above. Confidence intervals for the potential rate of screen-detected and interval cancers were adjusted for non-independent observations. Stata version 17.0 for Windows (StataCorp) was used to analyze the data.

## Results

Consensus was 8.8%, recall 3.2%, screen-detected cancer 6.1 per 1000 examinations, and interval cancer 1.7 per 1000 in the study sample, after independent double reading in a real screening setting (Table 2). With AI as one of the readers and a selection rate of the AI system equal to the average rate of positive assessment by the individual radiologists (5.8%), scenario 1 showed that 10.4% of the examinations would be selected for consensus. Recall in this scenario was 2.7% and the screen-detected cancer 5.9 per 1000 examinations. With a consensus rate of 14.2% in scenario 2 where all examinations with an AI score of 10 were selected for consensus, eight more screen-detected cancers were detected compared to scenario 1. The rate of screen-detected cancers was 5.9 per 1000 as for scenario 1. The consensus rate in scenario 2 resulted in the highest number of interval cancers where the prior screening examination was selected for consensus and the lowest potential rate of interval cancer.

**Table 2:** Consensus, recall, screen-detected (SDC), and interval cancer (IC) rate for 11 theoretical screen-reading scenarios with artificial intelligence and radiologists (R1 and R2). An AI score of 1 indicates the lowest probability of malignancy by the AI system, and 10 indicates the highest probability

Scenarios	Selection rate of AI and radiologists/setup based on AI score	Consensus, *n* (% of all examinations)	Recall, *n* (% of all examinations)	Screen-detected cancer, *n* (*n* per 1000)	Potential interval cancers detected as SDC among consensus and/or recall cases, *n* (*n* per 1000)^∞^	Potential rate of SDC (per 1000) with 95% confidence interval^§^	Potential rate of IC (per 1000) with 95% confidence interval^§^
	Independent double reading	10,790 (8.8)	3896 (3.2)	752 (6.1)	0	–	1.7 (*n* = 205)^€^
1	AI and R1 selects 5.8% each*	12,724 (10.4)	3357 (2.7)	721 (5.9)	77 (0.6)	6.5 (6.1–7.0)	1.0 (0.9–1.2)
2	AI selects 10.1% (AI score = 10) and R1 selects 5.8%*	17,394 (14.2)	3412 (2.8)	729 (5.9)	100 (0.8)	6.7 (6.3–7.2)	0.9 (0.7–1.0)
3	AI score 1–5: negative AI score 6–10: R1+R2	7955 (6.5)	3201 (2.6)	735 (6.0)	45 (0.4)	6.3 (5.9–6.8)	1.3 (1.1–1.5)
4	AI score 1–7: negative AI score 8–10: R1+R2	5894 (5.0)	2564 (2.1)	719 (5.8)	43 (0.3)	6.2 (5.8–6.7)	1.3 (1.1–1.5)
5	AI score 1–9: negative AI score 10: R1+R2	2805 (2.3)	1437 (1.2)	653 (5.3)	33 (0.3)	5.6 (5.2–6.0)	1.4 (1.2–1.6)
6	AI score 1–5: negative AI score 6–10: R1	5301 (4.3)	2669 (2.2)	651 (5.3)	28 (0.2)	5.5 (5.1–6.0)	1.4 (1.2–1.7)
7	AI score 1–5: R1 AI score 6–10: R1+R2	9683 (7.9)	3749 (3.1)	747 (6.1)	46 (0.4)	6.4 (6.0–6.9)	1.3 (1.1–1.5)
8	AI score 1–7: R1 AI score 8–10: R1+R2	8880 (7.2)	3614 (2.9)	746 (6.1)	45 (0.4)	6.4 (6.0–6.9)	1.3 (1.1–1.5)
9	AI score 1–5: negative AI score 6–7.5: R1 AI score 7.6–10: R1+R2	6934 (5.6)	3028 (2.5)	731 (5.9)	44 (0.4)	6.3 (5.9–6.8)	1.3 (1.1–1.5)
10	AI score 1–5: negative AI score 6–7.5: R1+R2 AI score 7.6–10: R1	6322 (5.1)	2842 (2.3)	655 (5.3)	29 (0.2)	5.6 (5.2–6.0)	1.4 (1.2–1.7)
11	3.2%*	-	3896 (3.2)	555 (4.5)	44 (0.4)	4.9 (4.5–5.3)	1.3 (1.1–1.5)

*5.8% mimics the average selection rate of the individual radiologists in the study sample, and 3.2% mimics the recall rate in the study sample after independent double reading

^∞^Number of interval cancers among consensus cases in Scenario 1–10. Number of interval cancers among cases that AI as a standalone system selected to be recalled in scenario 11

^§^If all IC with prior exams among consensus and/or recall cases were present at screening and detected as screen-detected and not interval cancers

^€^The actual rate and number of interval cancers in the study sample

In scenario 3, where examinations with an AI score of 1–5 were considered negative and examinations with an AI score of 6–10 were double read, consensus was 6.5%, recall 2.6%, and screen-detected cancer 6.0 per 1000 (Table 2). With the setup in scenarios 1, 2, and 3, the screen-reading volume would be reduced with 50% (Table 1). The potential reduction in volume in scenarios 4 and 5 is 70% and 90%, respectively, and a further drop in consensus and recall was observed at the expense of a reduced rate of screen-detected cancer (Table 2).

The highest rate of screen-detected cancer was observed for scenarios 7 and 8 where cases with low scores were read by one radiologist and cases with a high score were double read (Table 2). The rate was similar to independent double reading with five screen-detected cancers classified as negative by AI in scenario 7 and six in scenario 8. These scenarios have the lowest volume reduction, 25% and 35%, respectively (Table 1).

Comparing scenarios 3 and 9 where all examinations with an AI score of 1–5 were considered negative but the proportion of examinations with a score 6 or higher requiring double reading differed, 735 screen-detected cancers were detected in scenario 3 and 731 cancers in scenario 9 (Table 2). Despite small differences in cancer detection, scenario 3 has the potential to reduce the volume with 50% and scenario 9 with 63% (Table 1). The number of cases discussed at consensus was lower for scenario 9 compared to that for scenario 3 (Table 2).

In scenario 11, where AI as a standalone system selected a similar number of examinations to recall as independent double reading, the selected cases included 74% (555/752) of the screen-detected cancers (Table 2).

**Fig. 2:**
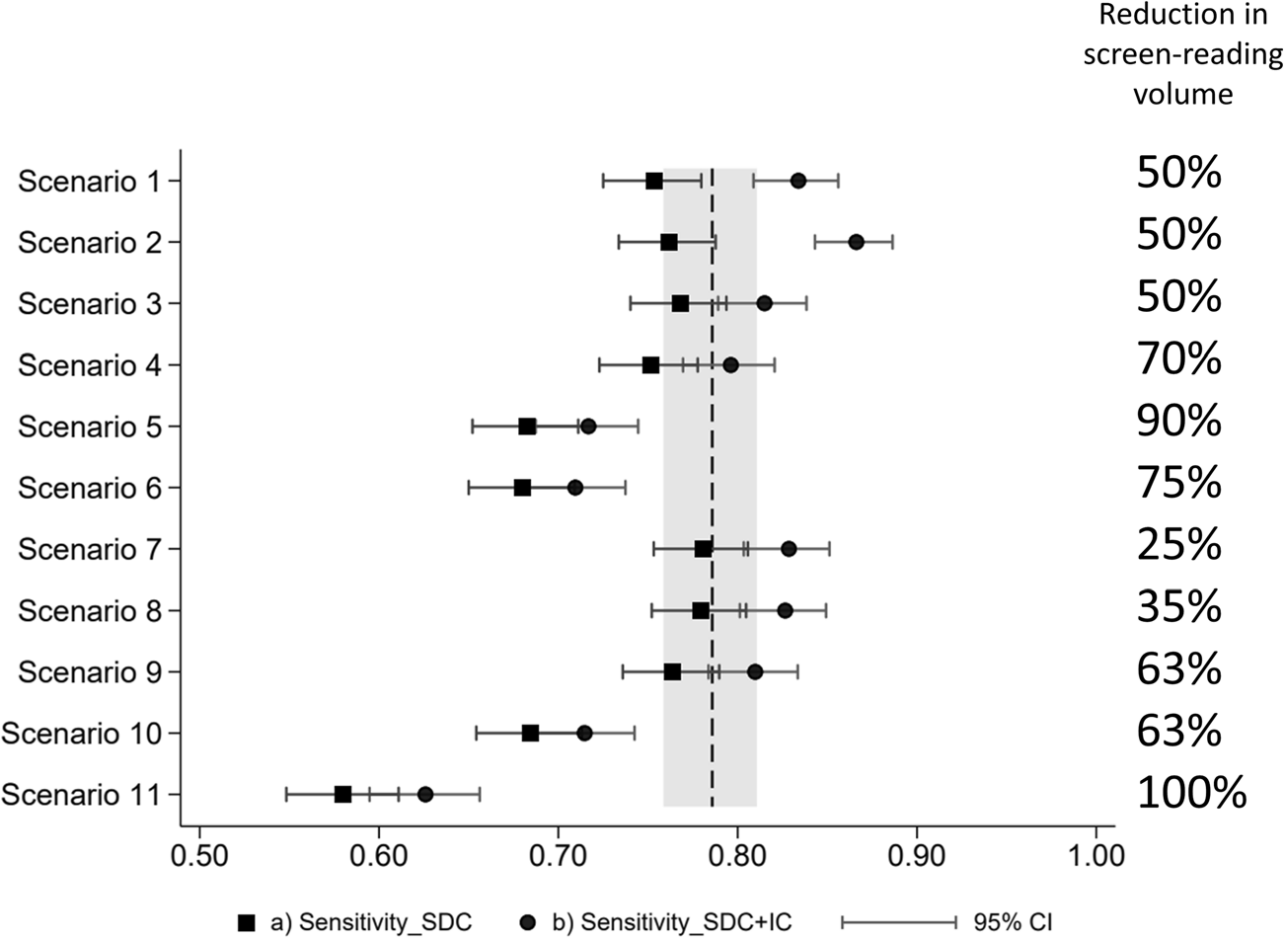
Sensitivity with 95% confidence interval (CI) for scenarios 1–11. The vertical dotted line with 95% CI bands represents the sensitivity after independent double reading where interval cancers (IC) are defined to be false negatives. In (a) only screen-detected cancers (SDC) are included as true positives and in (b) IC with prior screening examination selected for consensus were included in addition to SDC. Reduction in screen-reading volume for the different scenarios is presented to the right

Sensitivity for independent double reading was (78.6%, 95% CI: 75.9–81.1%). Including 95% CI for the sensitivity of the different scenarios with screen-detected cancers as true positive cases, estimated sensitivity and lower CI limits were above 70% for scenarios 1–4 and 7–9 (Fig. 2). In a prospective setting, we would expect the sensitivity to be somewhere between the two different sensitivity values (circle and square) for each scenario. For scenarios 1 and 2, the potenial rate of screen-detected cancers at the expense of lower interval cancer rate was higher than the observed screen-detected cancer rate after an independent double reading (Fig. 2, Table 2).

## Discussion

In this study using retrospective data from two breast centers in BreastScreen Norway, we estimated consensus, recall, and cancer detection rates for different theoretical scenarios of AI and radiologists in screen-reading. In scenarios 1 and 2, AI was used as one of two readers, and in scenarios 3–10, AI score was used to select examinations to be single or double read by radiologists. We found results for scenarios 1–4 and 7–9 to be promising as the recall rate is estimated to be reduced without observing a substantial decrease in the estimated cancer detection rate.

The estimated reduction in screen-reading volume was similar in a setting where AI and one radiologist read all examinations and selected cases for consensus (scenarios 1 and 2) and in a setting where examinations with an AI score of 1–5 were considered negative and 6–10 was read by two independent radiologists (scenario 3). There are pros and cons to both scenarios. From the same study sample, 23% of the screen-detected cancers were detected by only one of the two radiologists [18]. A proportion of the screen-detected cancers will not be selected for consensus in scenarios 1 and 2 despite using AI as the second reader. In scenario 3, there is no safety net of one radiologist interpreting mammograms with low AI scores, but in this study sample, a higher rate of screen-detected cancer was estimated for scenario 3. However, the result was not statistically different from that of scenarios 1 and 2, and the estimations were based on retrospective data. Results from a real screening setting might thus be different. Furthermore, histopathological tumor characteristics were shown to be less favorable for screen-detected cancers with higher versus lower AI scores. We consider scenarios where two radiologists interpret mammograms with high AI scores to reduce the risk of missing clinically significant cancers. For these reasons, setups where two radiologists interpret mammograms with higher AI scores might thus be preferred over AI as one of two readers. In addition, based on our previous publication of AI scores and cancer classification [18], we found a high proportion of interval cancers with a high AI score. As a result, some interval cancers might thus be detected earlier, as screen-detected if two radiologists read these examinations. This finding also supports double reading of mammograms with high AI scores.

The evidence of the potential AI has to reduce interval cancer rate at the expense of an increased (or non-inferior) screen-detected cancer rate is very limited. A research group from Sweden did an informed review of prior mammograms from 429 interval cancers. A total of 58% (83/143) of the interval cancers with an AI score of 10 were classified as a minimal sign or missed and correctly localized by the AI system [21]. This number corresponds to 19.3% (83/429) of all the interval cancers. In this study, we explored the potential of detecting interval cancers as screen-detected with the support of AI by considering all interval cancers selected for consensus as screen-detected. However, to achieve the estimated potential screen-detected cancer rate, we assumed the AI system to correctly localize the suspicious area and the radiologists to recall all the cancer cases. If the consensus agreed with the radiologists rather than the AI, our estimates were overestimated [22].

In scenarios 1 and 2, a proportion of the included screen-detected cancers were selected for consensus by the AI system and not R1. In the independent double reading setting, these cancers were detected due to the interpretation score by R2. The inclusion of these cases might overestimate the screen-detected cancer rate for scenarios 1 and 2 as it might not be reasonable to assume that all cases considered negative by a radiologist and positive by AI would be recalled after consensus. However, despite being selected for consensus by AI only, we chose to keep these cases as true positives as we expect some of these cases to be recalled due to the high AI score and the consensus with two or more radiologists. In scenario 2, a total of 9% of the 729 screen-detected cancers were selected for consensus by AI only. On the contrary, 10% were selected for consensus only by R1 and not AI. This means that 81% of the cancers were selected for consensus by both R1 and AI, and we consider the rate not to be substantially overestimated.

The optimal threshold of the AI score for selecting cases for single or independent double reading is difficult to decide to safely reduce volume. By substantially reducing the screen-reading volume, a higher consensus rate than the average in independent double reading might be an acceptable trade-off. How this will influence the subsequent recall rate and the costs are important aspects to consider. The recall rate among cases discussed at consensus might increase. However, the *overall* recall rate, i.e., recalled cases among all examinations, might not be influenced since AI defined cases that were discussed in consensus and recalled after independent double reading to be negative, i.e., not selected for consensus. However, we expect the examinations with a high score to be more time-consuming for the radiologists to screen-read compared to those with lower AI scores. The reduction in screening volume is thus not representing all aspects of workload.

If AI is used as decision support at consensus and not in the interpretation process, we expect the estimated consensus rates in this study to be about the same in a prospective setting with a similar setup. If AI scores and hotspots are made available for the radiologists during the individual interpretation process, the numbers might differ from our estimates since interpretations and conclusions are expected to be influenced differently than being presented to AI findings at consensus [22].

The strength of our study is the large study population from a real screening setting, including screen-detected as well as interval cancers. Rates of screen-detected and interval cancers vary among breast centers in BreastScreen Norway, and AI in screen-reading might support some centers more than others. During the first 20 years of screening in Norway, 1996–2016, the screen-detected cancer rate ranged from 4.4 to 6.7 [14]. Including mammograms from only two of 17 breast centers, and including women screened solely with Siemens equipment represent limitations of the study. We limited the number of scenarios to 11, but other setups of AI and radiologists might give additional perspectives. Other limitations were related to the assumptions and retrospective approach. We assumed that all cancer cases were selected and detected at recall, which might not be the case in a prospective screening setting [5, 23]. Furthermore, the thresholds mimicking results from independent double reading were derived from the study data and might not be representative of other breast centers. Also, the use of AI as a support in the interpretation process or in the consensus might influence the radiologists’ working process. Possible factors and how these factors are of influence for the interpretation in the startup and after some years need to be explored.

In conclusion, different scenarios of using AI as a support in mammographic screening have the potential to reduce screen-reading volume without reducing the rate of screen-detected cancers. Possible reduction of interval cancers and rates of false-positive results for the different scenarios have to be evaluated in prospective studies.

## Abbreviations

AI: Artificial intelligence
CI: Confidence interval
R1: Radiologist 1 (first reader)
R2: Radiologist 2 (second reader)

## Declarations

### Guarantor

The scientific guarantor of this publication is Solveig Hofvind.

### Statistics and biometry

One of the authors has significant statistical expertise (Marthe Larsen). In addition, no complex statistical methods were necessary for this paper.

### Study subjects or cohorts overlap

The same study sample with artificial intelligence results and screening information have been submitted to Radiology, but the research question differed (“Artificial intelligence for cancer detection in mammographic screening: retrospective evaluation of 122 969 screening examinations from a population-based screening program”, Larsen M, Aglen C et al, accepted in January 2022, *Radiology*, DOI: 10.1148/radiol.212381). The main focus in the Radiology article was to describe the overall performance of the AI system without exploring possible ways to use AI as a support/replacement in mammography screening.

### Methodology

• retrospective

• registry study

• multicenter study
